# Alloimmunization in Patients with Sickle Cell Disease in French Guiana

**DOI:** 10.1155/2015/812934

**Published:** 2015-02-02

**Authors:** Narcisse Elenga, Loic Niel

**Affiliations:** ^1^Pediatric Unit, Cayenne Hospital, rue des Flamboyants, BP 6006, 97306 Cayenne Cedex, French Guiana; ^2^French Guianese Blood Agency, Cayenne Hospital, rue des Flamboyants, BP 6006, 97306 Cayenne Cedex, French Guiana

## Abstract

This study in French Guiana assessed the frequency of alloimmunization to red cell antigens in sickle cell disease patients over 1995–2011 and identified the most common antibodies. A retrospective analysis of the transfusion history and medical records of 302 patients showed that 29/178 transfused patients had developed alloantibodies (16%). The most frequent alloantibodies were anti-LE1, anti-MNS1, anti-LE2, and anti-FY1 and were developed after transfusion of standard red cell units. The frequency of the clinically significant antibodies in this population of SCD patients was 11% (19/178). The antibodies found on those patients who had delayed hemolytic transfusion reaction were anti-K1, anti-FY1, and anti-MNS3. The strategies used to decrease alloimmunization in French Guiana are discussed.

## 1. Introduction

Sickle cell disease (SCD) is a major public health problem in French Guiana [1, 2]. In this French region located in South America, with 230,000 inhabitants, the standards of health are close to those of mainland France. French Guiana is also a crossroad for poor Caribbean and South American populations that emigrate there in search of better conditions of life. Infectious diseases and chronic diseases are both public health problems. Transfusion remains a major treatment in SCD management. The purpose of red blood cell transfusion (RBC) is to increase oxygen distribution in the tissues and to replace the rigid sickle cell shaped RBCs with healthy deformable RBCs. Yet, in French Guiana, since 2006, donation of blood products has stopped because of Chagas disease. Chagas disease (American trypanosomiasis) is caused by the protozoan* Trypanosoma cruzi*, mainly transmitted to humans by blood-sucking triatomine bugs (Hemiptera, Triatominae) but also transmitted by blood transfusion from infected donors and occasionally by transplacental mother-to-child transmission. It is an endemic disease in the large part of Latin America extending from Mexico to Argentina. In the majority of the cases, after an acute infection sometimes unapparent, the disease becomes chronic, which can be problematic, in case of blood donation.

In French Guiana, the overall prevalence of* T. cruzi *specific IgG was 0.5% [3–5]. All blood products used for transfusion in SCD patients come from Lille, in the north of France or Guadeloupe. Because they will frequently receive transfusions in their life, patients with SCD become exposed to RBCs alloantigens of donor units and alloimmunization. Alloimmunization in SCD has a reported incidence of 20% to 50% [6, 7]. Red cell alloimmunization is frequent because of the antigen disparities between patients of African descent and donors of European ancestry [8–11]. This study, therefore, aimed to estimate the frequency of alloimmunization in a cohort of SCD patients followed in the French Blood Agency of French Guiana from 1995 to 2011, in the particular context of French Guiana, where the blood units come mostly from Caucasian donors, and to describe the measures organized to decrease alloimmunization.

## 2. Methods

### 2.1. Study Population

302 SCD patients (151 males and 151 females) followed in the French Blood Agency of French Guiana from 1995 to 2011 were included. Data prospectively collected from the medical records monitoring transfusion included demographic and haematological characteristics. All transfused patients (*n* = 178) had received cross-matched red cell units at least compatible in the ABO, RH, and Kell systems.

### 2.2. Study Design

We reviewed the medical charts in the computerized blood transfusion database.

### 2.3. Ethical Consideration

Patients included in the database of the French Blood Agency gave informed consent for the use of their data. This data collection was approved by the Commission Nationale Informatique et Libertés (CNIL), a national committee that oversees research data.

### 2.4. Statistical Analysis

Data were analysed with STATA 10.0 (Stata Corp LP, College Station, TX, USA). We performed a descriptive analysis.

## 3. Results

Among these patients, 68% were homozygous for HbS, 24% had sickle-hemoglobin C, and 8% had HbS/*β*-thalassemia. The major ethnic origins were Creole (46%) followed by Haitian (27%) and Bushinenge (22%). The proportions of blood groups were group A: 21.80%, group B: 21.80%, group O: 51.50%, and group AB: 4.90%. The red cell blood transfusion was available for 178 patients (Hb SS), for 2494 red blood cell units. Among the sickle cell anemia patients who had received at least 1 transfusion, 29 had developed alloantibodies (16%). The most frequent alloantibodies were anti-LE1, anti-MNS1, anti-LE2, and anti-FY1 and were developed after transfusion of standard red cell units (Table 1). One patient had 4 alloantibodies while 3 patients had 3 alloantibodies. 12 patients had 2 alloantibodies and 13 patients had 1 alloantibody. There were 74 transfusions and 19 exchange transfusions. Transfusion was performed for acute chest syndrome (ACS) in 26 cases, cholecystectomy in 15 cases, splenectomy in 12 cases, severe infections in 12 cases, and stroke in 10 cases. The frequency of the clinically significant antibodies in this population of SCD patients was 11% (19/178). There were six cases of delayed hemolytic transfusion reaction (DHTR), in two children, two pregnant women, and two other adult SCD patients. The antibodies found on those patients who had DHTR were anti-K1, anti-FY1, and anti-MNS3.

## 4. Discussion

### 4.1. Prevalence of Alloimmunization

The prevalence of alloimmunization is high in French Guiana. Nevertheless in Martinique and in Guadeloupe, two other French overseas territories, this prevalence seems similar [12]. It is necessary to note that even if in these two territories the blood collection and distribution are realized on the spot, part of the blood units still come from metropolitan France, because the local production does not allow completely covering needs. Closer to French Guiana, in Brazil, where the prevalence of the Chagas disease in the general population is raised, the alloimmunization rate is 12.9% [13].

There is a basic assumption that since blood cannot be collected locally, use of blood mostly from French Caucasian blood donors is causing alloimmunization in the Guianese population due to ethnic/genetic differences. This assumption, although intuitive, has recently been scientifically refuted by an article on the SCD transfusion policy in a USA SCD center where it was shown that “ethnically” compatible blood in fact brings a high risk of alloimmunization [14].

### 4.2. Consequence of the Alloimmunization

The major consequence of alloimmunization is the delayed hemolytic transfusion reaction (DHTR), a severe and potentially life-threatening complication that is characterized by a hemolytic anemia of transfused as well as patients' own red blood cells. The diagnosis of DHTR can be difficult because the clinical features could be easily misinterpreted as a severe vasoocclusive crisis. In French Guiana, we noted 6 cases of DHTR that represent a rate of 3.4% (6/178). This frequency seems very high when compared with Brazil [15]. In Brazil, in spite of high prevalence of Chagas disease, blood collection in the general population has not been interrupted. So, blood units come from donors whose origin is close to those of patients. However, the prevalence of antibodies to* T. cruzi* among blood donors has decreased to 0.2% (2012) and in a serosurvey done in 2007 with children of 0–5 years old no single seropositive subject was detected [16] showing that Chagas is a vanishing disease and a minor problem concerning blood transfusion. No case of transmission of Chagas by blood transfusion was reported since the introduction of mandatory testing in the nineties. So, prevention of transfusion-transmitted (TT) Chagas in Brazil is based on serological testing and specific questions included in the predonation interview like the following: “have you ever seen a reduviid bug?”

French Guiana could follow the Brazilian model, which has proven itself. While waiting for the implementation of the blood donation in French Guiana, we recommend for SCD patients who require repeated transfusions extended antigen-matched RBC from Afro-Caribbean donors living in Guadeloupe and taking into account the patient's immunohematologic characteristics.

## 5. Conclusion

In French Guiana, the alloimmunization for the clinically significant antibodies in the SCD patients had a frequency of 11%. The prevention of TT Chagas based on serological testing and specific questions included in the predonation interview like “have you ever seen a reduviid bug?” as done in Brazil could be followed. This strategy for prevention of TT Chagas disease needs a strong political engagement of the sanitary authorities. This study also underlines the interest of selecting blood from Caribbean donors for all programmed transfusion or exchange transfusion.

## Figures and Tables

**Table 1:** Blood alloantibodies detected in 29 sickle cell disease patients who have developed alloantibodies.

Alloantibody	Number of patients
Anti-LE1	10
Anti-MNS1	6
Anti-LE2	5
Anti-FY1	5
Anti-RH2	4
Anti-RH3	3
Anti-JK1	3
Anti-MNS 3	2
Anti-K1	2
Anti-RH1	1
Anti-RH8	1
Anti-FY2	1
Anti-JK2	1
Anti-LE4	1
Anti-LU1	1
Anti-DB1	1
